# An Improved UWB Indoor Positioning Approach for UAVs Based on the Dual-Anchor Model

**DOI:** 10.3390/s25041052

**Published:** 2025-02-10

**Authors:** Zhengrong Xiang, Lei Chen, Qiqi Wu, Jianfeng Yang, Xisheng Dai, Xianming Xie

**Affiliations:** 1 School of Automation, Guangxi University of Science and Technology, Liuzhou 545006, China; zhengrongxiang0913@163.com (Z.X.); usrm6106@21cn.com (L.C.); mathdxs@163.com (X.D.); xxmxgm@163.com (X.X.); 2 School of Physics and Information Engineering, Guangxi Science & Technology Normal University, Laibin 546199, China; 3 China Electronic Product Reliability and Environmental Testing Research Institute, Guangzhou 510610, China; yangjeff2013@163.com

**Keywords:** unmanned aerial vehicle (UAV), ultra-wideband (UWB), unscented Kalman filter (UKF), indoor positioning, altitude fusion

## Abstract

Ultra-wideband (UWB) technology has been widely used for indoor positioning of UAVs due to its excellent range performance. The traditional UWB positioning system requires at least three anchors to complete 3D positioning. Reducing the number of anchors further means reducing the cost and difficulty of deployment. Therefore, this paper proposes a positioning model using only two anchors. In this model, the altitude of the UAV is measured by a rangefinder. Then, the position of the UAV is projected onto the horizontal plane, converting 3D positioning into 2D positioning. The rangefinder’s range accuracy is higher than that of the UWB, which is beneficial for improving 3D positioning accuracy. In addition, an altitude fusion method of integrating rangefinder and barometer data is designed to realize the switching of altitude data and barometer calibration to solve the problem of obstacles under the UAV affecting the altitude measurement. On this basis, the multi-sensor data fusion algorithm based on a dual-anchor positioning model is designed to improve positioning accuracy, and the data of the UWB, rangefinder, barometer, and accelerometer are fused by the unscented Kalman filter (UKF) algorithm. The positioning simulation and experiment show that the positioning accuracy of the dual-anchor model is generally higher than that of the three-anchor model, with decimeter-level positioning accuracy.

## 1. Introduction

At present, Unmanned Aerial Vehicles (UAVs) have been employed extensively across a range of industries. The classification of UAVs encompasses fixed-wing UAVs, multi-rotor UAVs, and other variants [1]. The multi-rotor UAV, in particular, has the function of vertical take-off and landing, thus offering significant advantages in domains such as photography, mapping, and line inspection [2,3]. With the development of UAV technology, UAVs have been widely used in indoor environments, such as transporting goods, recording instrument data, and collecting cargo information. These functions can be carried out depending on a UAV that has an accurate and stable positioning system. Therefore, a variety of schemes are proposed and applied to meet the positioning requirements in different scenarios. Mainstream indoor positioning technologies can be divided into two categories. One is vision-based and includes Motion Capture Systems (MCSs) [4,5], Simultaneous Positioning and Mapping (SLAM) [6,7,8], and LiDAR [9,10,11]. The other is based on time-of-flight (TOF) signals and includes ultra-wideband (UWB) positioning, WiFi positioning, and Bluetooth positioning.

The UWB ranging chip serves as the foundation for achieving UWB positioning. Currently, the commonly used UWB chips are DW1000 and DW3000 [12,13], boasting a ranging accuracy of 10 cm. Additionally, the DW3000, when utilizing dual antennas, can measure horizontal angles with a measurement range of 120° and an accuracy of 5°. UWB positioning technology is a method of calculating the coordinates of a tag by measuring the distance between the tag and anchors and then utilizing the triangle side–length relationship to calculate the coordinates. Its positioning accuracy is typically within a range of 20 cm [14,15], which is widely used in indoor positioning. Theoretically, UWB-based 3D positioning requires only three anchors. Some researchers have discussed increasing the number of anchors or tags to further improve the positioning accuracy of the UWB. Queralta et al. [16] provided open-source data by running seven sets of tests on a platform they had built with different numbers of anchors and tags. Aiming at position estimation for coplanar base station scenarios, Zhou et al. [17] proposed an optimization algorithm based on Newton’s iterative method, which resulted in an average improvement of 63.54% in test point accuracy. Nevertheless, the height at which anchors can be deployed indoors is constrained, which resulted in a significant reduction in the positioning accuracy of the *z*-axis in comparison to that of the *xy*-axis. To improve the *z*-axis positioning accuracy, Euiho et al. [18] used the method of searching for the optimal geometry of the UWB positioning network generated from 10 anchors. In addition to increasing the number of anchors, fusion algorithms can also be used to fuse other sensor data to improve positioning accuracy. Zeng et al. [19] proposed a UAV landing position method with dual UWB tags fused with an inertial measurement unit (IMU). Li et al. [20,21,22] combined the distance information measured by a UWB signal with the acceleration information measured by IMUs and used the extended Kalman filter (EKF) to fuse the data, which significantly improved the positioning accuracy. Guo et al. [21] reduced the maximum positioning error to 0.2 m in a 7 m × 7 m positioning area, whereas Guosheng et al. [22] specifically focused on clock bias errors, used a third-order polynomial fitting method to compensate for the remaining distance errors in the symmetric dual-side ranging method (SDS-TWR), and then proposed an error complementary fusion method to achieve sub-decimeter positioning accuracy. Nonetheless, the linear approximation method employed by EKF is not universally applicable, particularly in instances of substantial nonlinearity or sudden fluctuations in noise. Conversely, You et al. advocated that an unscented Kalman filter (UKF) can be utilized to estimate the system’s state with greater efficacy [23,24]. In addition, some researchers have been discussing integrating UWB with other position technologies, such as GNSS, LiDAR, and vision [25,26,27]. It is evident that augmenting the number of sensors to enhance the positioning accuracy is a highly efficacious approach.

The UWB positioning method studied in References [16,17,18,19,20,21,22,23,24,25,26,27] uses three or more anchors. Since the location of the anchors must be known and deployed in advance, they are usually placed manually using measuring instruments. In addition, the normal operation of anchors depends on a constant power supply (whether connected to the grid or mobile power), which complicates the deployment of the anchor. Therefore, reducing the number of anchors required for UAV positioning can reduce cost and deployment effort, and further improve the environmental adaptability of UWB-based UAVs, because less ground space is required for anchor deployment. The authors of [28,29] proposed two robot positioning algorithms, both of which use only one UWB anchor combined with an inertial sensor. Although they are only for mobile robots at fixed altitudes, they have a reference value for UAVs. Among them, Cao et al. [28] assumed that the robot moves at a constant speed. Although this fits most robotic application scenarios, it cannot operate in a variable-speed environment, which will have many limitations, especially in the UAV application scenario, leading to the risk of collision or even falling UAVs. Zhang et al. [29] used the inertial sensor and UWB to form a heterogeneous sensor, which observes the displacement difference from the inertial sensor and the difference in the radial distance of the UWB at two different times to locate the robot. However, the accumulated error of the walking distance of INS leads to unsatisfactory positioning accuracy. Zhou et al. [30] constructed a factor graph using UWB measurements and measurement increments and proposed an adaptive confidence region using UWB measurements and measurement increments as observation vectors. The iterative solution of the positioning coordinate reduced the original positioning accuracy from 0.84 m to 0.21 m. However, constructing the factor map is not easy, and the factor map of each positioning network needs to be rebuilt. As the basic positioning unit, to expand multiple positioning networks and improve the interference of the non-line-of-sight (NLOS) environment, more factor maps need to be constructed, which further increases the working time of positioning facilities. These positioning methods that use single anchors are employed for robot positioning, which is fundamentally 2D positioning on the horizontal plane. However, the flight of a UAV depends on 3D positioning; these methods cannot be directly applied to the indoor positioning of a UAV. Should the anchor be capable of providing multiple data points concurrently, the radar, for instance, could furnish the distance, velocity, and 3D angle of the target. The utilization of these data points would facilitate the precise calculation of the target’s coordinates [31]. However, radar is suitable for outdoor open environments or scenarios requiring long-range detection, but it is not suitable for indoor positioning. Additionally, UWB can only provide the distance and low-precision horizontal angle of the target, and it is still a very challenging task to achieve 3D positioning with only one UWB anchor.

In summary, the augmentation of sensor numbers can enhance the precision of positioning. However, this is concomitant with an escalation in financial expenditure. Utilizing a solitary anchor has the advantage of simplifying deployment; nevertheless, it is challenging to attain 3D positioning. This paper seeks to improve the UWB positioning model from a different perspective to reduce the number of anchors while maintaining good 3D positioning accuracy. It is generally accepted that, to calculate 3D coordinates, at least three parameters are required mathematically. In the context of traditional UWB positioning, these parameters are typically UWB measurements, which are subject to about 10 cm errors. The incorporation of additional high-precision sensors to substitute for a proportion of the UWB measurement distance is a strategy that has the potential to enhance positioning accuracy. During flight, UAVs utilize high-precision laser rangefinders to measure flight altitude, and it is possible to replace one of the UWBs with the rangefinder, presenting an opportunity for applying an improved positioning model. The improved model is designated the dual-anchor model in this paper, which employs the triangle side–length relationship to calculate the positioning position. The primary contribution of this paper is to resolve the issues encountered in the application of the dual-anchor model on UAVs, as well as utilizing multiple sensors to further enhance the positioning accuracy, as follows:

1. A dual-anchor positioning model designed for the indoor environment of UAVs is proposed, which not only reduces the number of anchors but also achieves high positioning accuracy. To comprehensively study the performance of the dual-anchor positioning model, the positioning formula, the maximum positioning error formula, and the positioning standard deviation formula are derived, and the positioning simulation is carried out.
2. A fusion method for measuring the flight altitude of a UAV was designed, which integrates the data of a laser rangefinder and barometer to solve the problem of obstacles under the UAV affecting the altitude measurement when using the dual-anchor positioning model.
3. A position-filtering algorithm was designed to be applied in the dual-anchor model. Based on the UKF algorithm, the data of the UWB, laser rangefinder, barometer, and accelerometer were fused to further improve the positioning accuracy of the dual-anchor positioning model.
4. A quadcopter was used as a test platform, and a series of simulations and experiments, including static positioning experimentation, dynamic positioning experimentation, and comprehensive experimentation, were conducted to assess the performance of the dual-anchor positioning model.

## 2. UWB Positioning Model

This section commences with a concise overview of the conventional three-anchor positioning model. Then, the positioning principle of the dual-anchor positioning model is introduced in detail, followed by a derivation of the positioning and positioning error equations and, finally, an investigation into potential avenues for enhancing the adaptability of the dual-anchor positioning model.

### 2.1. Traditional Three-Anchor Positioning Model

**Figure 1:**
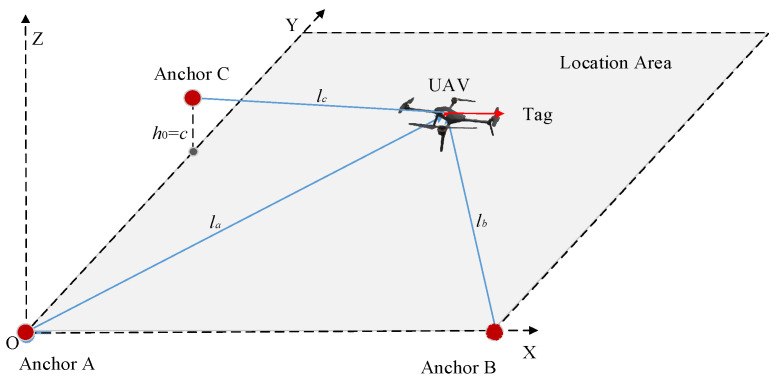
Three-anchor positioning model.

The traditional three-anchor positioning model is depicted in Figure 1. There are numerous potential deployment strategies for UWB anchors, and for ease of comparative analysis with the dual-anchor positioning model, UWB anchors A, B, and C are deployed at coordinates A(0, 0, 0), B(*a*, 0, 0), and C(*b*, 0, *c*), respectively. UWB tags are equipped on the UAV for measuring the straight-line distance between the UAV and each anchor, noted as *l_a_*, *l_b_*, and *l_c_*, respectively.

Assuming that the UAV coordinates are P(*p_x_*, *p_y_*, *p_z_*), the following relationship exists:

(1) $$\left\{\begin{array}{l} l_{a}=\sqrt{p_{x}^{2}+p_{y}^{2}+p_{z}^{2}}\\ l_{b}=\sqrt{{\left(a-p_{x}\right)}^{2}+p_{y}^{2}+p_{z}^{2}}\\ l_{c}=\sqrt{p_{x}^{2}+{\left(b-p_{y}\right)}^{2}+{\left(c-p_{z}\right)}^{2}} \end{array}\right.$$

The transformation of the above Equation (1) yields the following equation:

(2) $$\left\{\begin{array}{c} p_{x}=\frac{l_{b}^{2}-l_{a}^{2}-a^{2}}{-2a}\\ p_{y}=S-\frac{c}{b}z\\ p_{z}=\left[\frac{Sc}{b}\pm \sqrt{{\left(\frac{Sc}{b}\right)}^{2}-(\frac{c^{2}}{b^{2}}+1)\cdot (M^{2}+x^{2}{-l}_{a}^{2})}\right]/(\frac{c^{2}}{b^{2}}+1)\\ S=\frac{l_{a}^{2}-l_{c}^{2}+b^{2}+c^{2}}{2b} \end{array}\right.$$

### 2.2. Dual-Anchor Positioning Model

**Figure 2:**
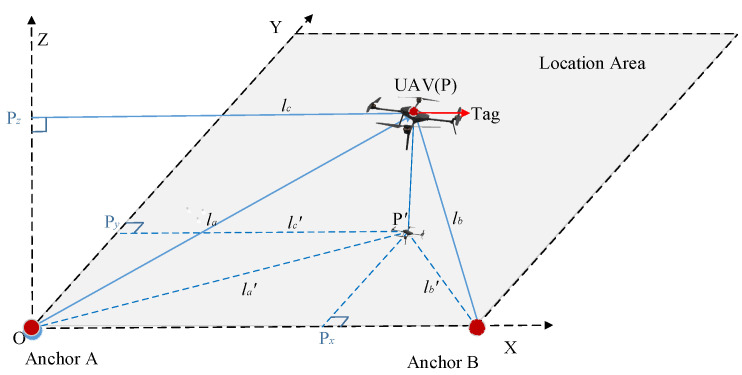
Dual-anchor positioning model.

The dual-anchor positioning model is depicted in Figure 2. The deployment of UWB anchors A and B on the ground, in conjunction with the utilization of a UWB tag and a laser rangefinder equipped on the UAV, enables the measurement of three pivotal distances: the straight-line distance between the UAV and anchor A designated as *l_a_*, that between the UAV and anchor B designated as *l_b_*, and the distance from a ground designated as *h*. The position of the UAV is then projected onto the horizontal plane, transforming the 3D position into a 2D position. The horizontal distance of the UAV from anchor A is designated as *l_a’_*, which can be calculated using the Pythagorean theorem. Together with *l_a_* and *h*, it gives the length of the side of the right-angled triangle named APP’. Similarly, the horizontal distance, denoted as *l_b’_*, between the UAV and anchor B can be calculated. The distance between anchors A and B is denoted *b*, together with *l_a’_* and *l_b’_*, forming the length of the side of the right-angled triangle ABP’. Given the coordinates of anchors A and B, the horizontal direction coordinates can be calculated as *p_x_* and *p_y_*. Then, the UAV’s coordinates in the vertical direction can be calculated as *p_z_*, which represents the UAV’s altitude *h*. Consequently, the 3D coordinates of the UAV are successfully obtained.

In the established 3D coordinate system, the *xy*-plane is parallel to the ground, the *z*-axis is perpendicular to the ground, and the positioning area is within the first quadrant of the coordinate system. Anchors A and B are deployed at coordinates A(0,0,0) and B(*a*,0,0), respectively. The UAV flies within the designated area, and the vertical coordinate *p_z_* is determined to be the altitude of the UAV from the ground, named *h*.

(3) $$p_{z}=h$$

The following steps are followed to calculate the horizontal direction coordinates *p_x_* and *p_y_*.

(1) The coordinates of the UAV, named P, are projected onto the *xy*-plane, designated as P’(*p_x_*, *p_y_*, 0). The projections of the lines, named AP and BP, onto the *xy*-plane are represented by AP’ and BP’, respectively. In the right-angled triangle PAP’, AP’ denotes a right-angled edge, the length of which is designated as *l_a’_*. Similarly, in the right-angled triangle PBP’, BP’ denotes a right-angled edge, the length of which is defined as *l_b’_*. *l_a’_* and *l_b’_* can be determined through the following equation:

(4) $$l_{a}^{’}=\sqrt{l_{a}^{2}-h^{2}},l_{b}^{’}=\sqrt{l_{b}^{2}-h^{2}}$$

Similarly, in the right-angled triangle PBP’, BP’ denotes a right-angled edge, the length of which, designated as *l_b’_*, can be determined through the following equation:

(2) The projections of the projected point P’ on the *x-* and *y*-axes are *p_x’_* and *p_y’_*, respectively. In the right-angled triangle AP’P*_x’_*, AP’ denotes a right-angled edge, the length of which is designated as *l_a’_*. Similarly, in the right-angled triangle P’P*_x’_*B, BP’ denotes a right-angled edge, the length of which is defined as *l_b’_*, and can be determined through the following equation:

(5) $$l_{a}^{’}=\sqrt{p_{x}^{2}+p_{y}^{2}},l_{b}^{’}=\sqrt{{\left(a-p_{x}\right)}^{2}+p_{y}^{2}}$$

(3) Equations (3) to (5) should be combined to derive the formula for the horizontal direction coordinates *p_x,_ p_y_*, and *p_z_*, which can be expressed as follows:

(6) $$\left\{\begin{array}{l} p_{x}=\frac{l_{a}^{2}-l_{b}^{2}+a^{2}}{2a}\\ p_{y}=\sqrt{l_{a}^{2}-h^{2}-{\left(\frac{l_{a}^{2}-l_{b}^{2}+a^{2}}{2a}\right)}^{2}}\\ p_{z}=h \end{array}\right.$$

### 2.3. Positioning Error Estimation

The function of the positioning model, as discussed in Section 2.1 and Section 2.2, is to transform measurements M(*m*_1_, *m*_2_, *m*_3_) into position coordinates P(*p_x_*, *p_y_*, *p_z_*) through the application of transformation function *T*. For the three-anchor positioning model, the conversion function is Equation (2), and the measurements are M(*l_a_*, *l_b_*, *l_c_*); for the dual-anchor positioning model, the conversion function is Equation (6) and the measurements are M(*l_a_*, *l_b_*, *h*). The increment in the conversion function can be expressed as a complete differentiation of this function. For calculating the positioning error, it is feasible to replace the measurement error of the individual measurements with a direct approximation of the individual components. This is demonstrated in the following equation:

(7) $$dp_{j}=\sum_{i=1}^{3}\frac{\partial T}{\partial m_{i}}dm_{i}\overset{dm_{i}=\Delta m_{i},dp=\Delta p}{\Rightarrow }\Delta p_{j}\approx \sum_{i=1}^{3}\frac{\partial T}{\partial m_{i}}\Delta m_{i},j=x,y,z$$

For the three-anchor positioning model, the expression of the conversion function is complex and challenging to analyze for errors. Therefore, the following equation, which can be obtained by differentiating both sides of the equal sign in Equation (1) simultaneously, is used to analyze the positioning errors:

(8) $$\left\{\begin{array}{l} dl_{a}=\frac{p_{x}}{l_{a}}dp_{x}+\frac{p_{y}}{l_{a}}dp_{y}+\frac{p_{z}}{l_{a}}dp_{z}\\ dl_{b}=\frac{p_{x}-a}{l_{b}}dp_{x}+\frac{p_{y}}{l_{b}}dp_{y}+\frac{p_{z}}{l_{b}}dp_{z}\\ dl_{c}=\frac{p_{x}}{l_{c}}dp_{x}+\frac{p_{y}-b}{l_{c}}dp_{y}+\frac{p_{z}-c}{l_{c}}dp_{z} \end{array}\right.$$

Written in vector form and approximated using Equation (7), the results are calculated as follows:

(9) $$\Delta M_{1}=B_{1}\Delta P=\left[\begin{array}{ccc} \frac{p_{x}}{l_{a}} & \frac{p_{y}}{l_{a}} & \frac{p_{z}}{l_{a}}\\ \frac{p_{x}-a}{l_{b}} & \frac{p_{y}}{l_{b}} & \frac{p_{z}}{l_{b}}\\ \frac{p_{x}}{l_{c}} & \frac{p_{y}-b}{l_{c}} & \frac{p_{z}-c}{l_{c}} \end{array}\right]\Delta P$$

where $M_{1}={\left[\begin{array}{ccc} l_{a} & l_{b} & l_{c} \end{array}\right]}^{T},P={\left[\begin{array}{ccc} p_{x} & p_{y} & p_{z} \end{array}\right]}^{T}$. The position error estimate is obtained using the pseudo-inverse method, as follows:

(10) $$\Delta P={\left(B_{1}^{T}B_{1}\right)}^{-1}B_{1}^{T}\Delta M_{1}$$

From matrix **B**_1_, it can be observed that when *l*_a_, *l*_b_, or *l*_c_ is close to 0, this results in an infinite positioning error. Therefore, to reduce this error to a level below the desired value, it is necessary to maintain a certain distance between the effective positioning area and the anchors.

In accordance with the dual-anchor positioning model, the transformation function is also fully differentiated, as demonstrated by the following equation:

(11) $$\left\{\begin{array}{l} dp_{x}=\frac{l_{a}}{a}dl_{a}-\frac{l_{b}}{a}dl_{b}\\ dp_{y}=\frac{1}{p_{y}}\left[l_{a}(1-\frac{p_{x}}{a})dl_{a}+\frac{p_{x}l_{b}}{a}dl_{b}-p_{z}dh\right]\\ dp_{z}=dh \end{array}\right.$$

Written in vector form and approximated using Equation (7), the results are calculated as follows:

(12) $$\Delta P=B_{2}\Delta M_{2},B_{2}=\left[\begin{array}{ccc} \frac{l_{a}}{a} & -\frac{l_{b}}{a} & 0\\ \frac{(a-p_{x})l_{a}}{p_{y}a} & \frac{p_{x}l_{b}}{p_{y}a} & -\frac{p_{z}}{p_{y}}\\ 0 & 0 & 1 \end{array}\right]$$

where $M_{2}={\left[\begin{array}{ccc} l_{a} & l_{b} & h \end{array}\right]}^{T}$. From matrix **B**_2_, it can be observed that since *a* acts as the denominator, a small value of *a* will lead to a significant positioning error. Consequently, to ensure the measurement error remains below the desired threshold, the distance between the two anchors must exceed a specific value *a*_min_. Since *p_y_* also serves as the denominator, a small value of *p_y_* will lead to a significant positioning error along the *y*-axis. Therefore, to ensure the *y*-axis error remains below the desired threshold, the effective positioning area should maintain a certain distance from the *y*-axis.

Subsequently, the standard deviation of the dual-anchor positioning model is analyzed. The functional relationship between the input and output of the proposed dual-anchor positioning model is smooth, continuous, and differentiable; then, for a small change in the field *m*_0_, the function *T* can be approximated by the first-order term of the Taylor series (i.e., the higher-order terms are ignored) [32] as follows:

(13) $$\sigma_{p}=\sqrt{\sum_{i=1}^{n}{\left(\frac{\partial f}{\partial m_{i}}\right)}^{2}\sigma_{m_{i}}^{2}+2\sum_{i=1}^{n}\sum_{j=i+1}^{n}\left(\frac{\partial f}{\partial m_{i}}\right)\left(\frac{\partial f}{\partial m_{j}}\right)Cov(m_{i},m_{j})}$$

where $Cov(m_{i},m_{j})=E\left[\left(m_{i}-E\left[m_{i}\right]\right)\left(m_{j}-E\left[m_{j}\right]\right)\right]$ is the covariance, where *E* is the statistical expectation value, and if the *m_i_* values are not correlated, the last equation can be simplified to the following standard equation for error propagation [32,33] as follows:

(14) $$\sigma_{p}=\sqrt{\sum_{i=1}^{n}{\left(\frac{\partial f}{\partial m_{i}}\right)}^{2}\sigma_{m_{i}}^{2}}$$

Because the physical quantities measured by the dual-anchor positioning model are all mutually independent of one another, the standard deviation of the dual-anchor model can be estimated on the basis of Equation (11).

(15) $$\left\{\begin{array}{l} \sigma_{p_{x}}=\frac{1}{a}\sqrt{{l_{a}}^{2}\sigma_{l_{a}}^{2}+{l_{b}}^{2}\sigma_{l_{b}}^{2}}\\ \sigma_{p_{y}}=\frac{1}{p_{y}}\sqrt{l_{a}^{2}{\left(1-\frac{p_{x}}{a}\right)}^{2}\sigma_{l_{a}}^{2}+\frac{p_{x}l_{b}}{a}\sigma_{l_{b}}^{2}-p_{z}\sigma_{h}^{2}}\\ \sigma_{p_{z}}=\sigma_{h} \end{array}\right.$$

In accordance with the distance formula between two points, the standard deviation of the distance estimate of each point can be calculated as follows:

(16) $$\sigma_{p_{d}}=\sqrt{\frac{{p_{x}}^{2}\sigma_{p_{x}}^{2}+{p_{y}}^{2}\sigma_{p_{y}}^{2}+{p_{z}}^{2}\sigma_{p_{z}}^{2}}{p_{x}^{2}+p_{y}^{2}+p_{z}^{2}}}$$

Then, the standard deviation can be calculated by combining Equation (14).

### 2.4. Multi-Area Positioning of Dual-Anchor Model

**Figure 3:**
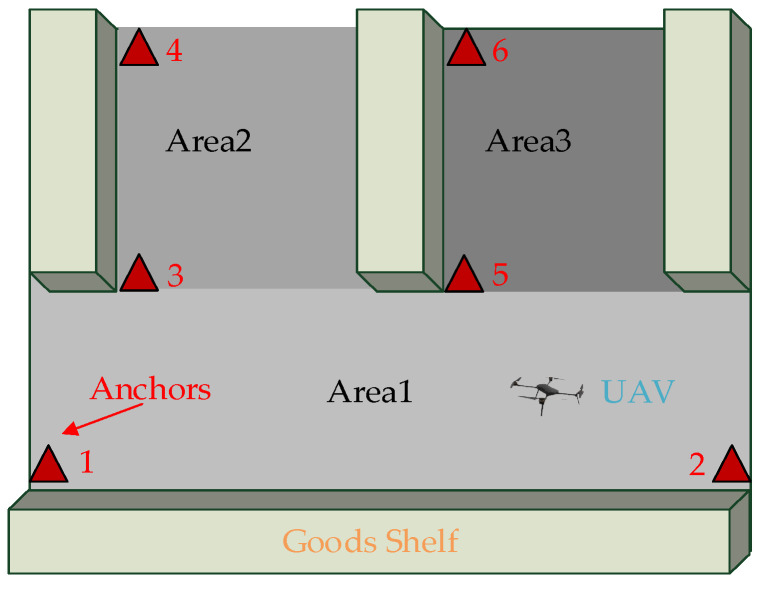
The schematic diagram of multi-area positioning.

The dual-anchor model is a fundamental unit for achieving location in a single area. However, multi-area positioning is more prevalent in practical requirements. For instance, in the cargo information acquisition system implemented by UAVs, as illustrated in Figure 3, a total of six anchors are deployed in the three positioning areas, designated as Area 1, Area 2, and Area 3. Additionally, two anchors are deployed in each area to achieve multi-area positioning. In the event of the traditional multi-anchor model being utilized, a minimum of 9 models will be required, representing a minimum increase of 33% in comparison to the dual-anchor model. In certain areas, the utilization of more than two anchors for distance measurement is permissible. As illustrated in the accompanying Figure 3, the position of the UAV can measure the distance of anchors 1–4, 5, and 6 simultaneously, thereby acquiring 10 sets of positioning data. The subsequent processing of these data is facilitated by a data fusion algorithm, a process that serves to enhance the positioning accuracy. It is evident that the utilization of dual anchors offers distinct advantages in the context of multi-area positioning applications.

### 2.5. A Non-Negligible Problem of the Dual-Anchor Positioning Model

The UAV altitude is measured by a laser rangefinder in the dual-anchor positioning model, where the operational principle of a laser rangefinder is using the flight time of the laser between the rangefinder and the object being measured to calculate the distance to the object. The presence of other objects beneath the UAV becomes an obstacle to altimetry, affecting the measurement of the UAV’s flight altitude. In this instance, differential barometric altimetry, which is not impeded by obstacles, can be employed as a viable solution.

The fundamental principle underlying differential barometric altimetry is as follows: by the laws of physics, the atmospheric pressure and temperature decrease with increasing altitude. Therefore, the difference in atmospheric pressure between two points at disparate elevations can be measured by the barometer and put into the conversion formula to calculate the altitude of the object in question. The process of altitude measurement is as follows: First, the reference air pressure *P*_0_ and the reference air temperature *T*_0_ are measured at the moment of UAV take-off. Subsequently, air pressure *P* and air temperature *T* are measured in real-time during the flight process. Finally, altitude *h* is then calculated by substituting into the following formula:

(17) $$h=18,410(1+\frac{T_{m}}{273.15})\mathrm{lg}\frac{P_{0}}{P},T_{m}=\frac{T_{0}+T}{2}$$

Barometer altimetry has a high measurement accuracy; taking the barometer ICP-10101 [34] produced by TDK as an example, its measurement accuracy is 8.5 cm. Furthermore, the accuracy of the system can be enhanced by integrating the acceleration data from the UAV.

Remarks: Barometric altimetry is subject to an intrinsic limitation. This limitation is a consequence of the gradual drift in the reference barometric pressure and temperature during the flight of a UAV, which results in a cumulative measurement error that becomes significant over extended flight durations.

## 3. Work Related to Positioning

**Figure 4:**
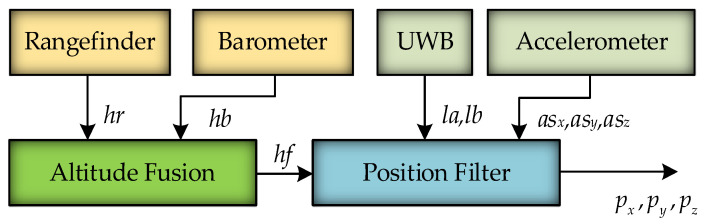
The structure of the positioning system.

The structure of the positioning system based on the dual-anchor positioning model is depicted in Figure 4. In this system, the laser rangefinder measurements are designated as *h_r_*, while the barometer measurements are defined as *h_b_*. *h_r_* and *h_b_* are employed as inputs of the altitude fusion, while the fused altitude data, specified as *h_f_*, serve as the output. The rangefinder outputs altitude data *h_r_*, the barometer outputs altitude data *h_b_*, and the optimal altitude estimation *h_f_* is obtained after altitude fusion. The UWB measurements of anchors A and B are designated as *l_a_* and *l_b_*, respectively.

In contrast, the accelerometer measurements in the *x*, *y*, and *z* directions are denoted as *as_x_*, *as_y_*, and *as_z_*, respectively. *h_f_* together with *l_a_*, *l_b_*, *as_x_*, *as_y_*, and *as_z_* are employed as inputs of the position filter, while an estimate of the UAV position, designated as *p_x_*, *p_y_*, and *p_z_*, serves as the output. The implementation of the system mainly employs altitude fusion methods and position-filtering algorithms based on the EKF.

### 3.1. Altitude Fusion Method

To address the shortcomings of the dual-anchor model mentioned in Section 2.4 after using a laser rangefinder or barometer for altitude measurement, the following altitude fusion method is proposed: When there are no obstacles below the UAV, *h_r_* is considered the exact flight altitude of the UAV and is used for localization purposes. At the same time, *h_b_* is calibrated using *h_r_*. Conversely, when an obstacle is present below the UAV, *h_b_* is utilized for positioning, and the calibration of the barometer is paused. To prevent barometer errors from exceeding the maximum value, the UAV needs to fly to an obstacle-free area for barometer calibration within a specified time frame. This method comprises two principal components: altitude switching and altitude calibration.

In terms of altitude switching, a critical aspect lies in accurately detecting the presence of obstacles beneath the UAV. The fulfillment of the following conditions indicates that there are no obstacles beneath the UAV, thereby enabling the altitude fusion method to output data from the laser rangefinder, as follows:

(1) The discrepancy between the laser rangefinder and barometer, designated as Δ*h*_1_, is less than the threshold value *h*_1_. This condition is employed for the rapid identification of elevated objects, such as boxes or tables, as follows:

(18) $$\Delta h_{1}=h_{b}-h_{r}<h_{1}$$

(2) Smoothing is applied to Δ*h*_1_ to improve measurement resolution, and the average value of Δ*h*_1_ over *n* sampling periods, denoted as Δ*h*_2_, is smaller than the threshold value *h*_2_. This condition is employed for the purpose of detecting obstacles with lower heights or those exhibiting slow changes, such as books or slopes, as follows:

(19) $$\Delta h_{2}=\frac{\sum \Delta h_{1}}{n}<h_{2}$$

In terms of altitude calibration, the measured altitude of the barometer is calibrated using the altitude measurement obtained from the laser rangefinder. It can be posited that the *h_b_* value derived from the barometer represents the true altitude *h* in conjunction with the error term *e_b_*. Similarly, the *h_r_* value obtained from the laser rangefinder can be conceptualized as comprising *h* and the error term *e_r_*, as follows:

(20) $$h_{b}=h+e_{b},h_{r}=h+e_{r}$$

The barometer’s measurement error varies over time, with a maximum error of up to 20 cm within 10 min. By contrast, the maximum error of the laser rangefinder is significantly smaller, at only 2 cm, and does not vary over time. Consequently, the laser rangefinder can be used to calibrate the barometer. The calibration method aims to obtain the calibration value, represented by the symbol △*e*, by subtracting *h_r_* from *h_b_*, as follows:

(21) $$\Delta e=h_{b}-h_{r}=e_{b}-e_{r}$$

To obtain the calibrated measured altitude following calibration, which is named *h_b1_*, Δ*e* should be subtracted from *h_b_*:

(22) $$h_{b1}=h_{b}-\Delta e=h+e_{r}$$

Since *e_r_* is much smaller than *e_b_*, the accuracy of the barometer measurements is substantially enhanced.

Given the interdependence of altitude switching and altitude calibration, performing barometric calibration under incorrect conditions may cause the fusion method to fail. Therefore, altitude calibration should only be conducted under stricter conditions. For the fusion method to proceed with altitude calibration, the following three conditions must be met simultaneously:

(1) Δ*h*_2_ must be less than the threshold value *h*_3_, which must be less than *h*_2_, which ensures that there are no obstacles beneath the UAV:
  (23) $$\Delta h_{2}<h_{3},h_{3}<h_{2}$$
(2) The variance *D*_h_ of the barometer within a unit of time must be less than the threshold *h*_4_, which ensures that the UAV maintains a constant altitude during a period, thereby enhancing the accuracy of the barometer measurements:
  (24) $$D_{h}<h_{4}$$
(3) To circumvent the potential issue of rapid calibration, which leads to the failure to detect smaller objects, the calibration period is set to several sampling periods. Calibration is only permitted when the calibration moment is reached. Denoting the current moment as *t*, the last calibration moment as *t*_1_, and the calibration period as Δ*t*, the following conditions need to be satisfied for altitude calibration:
  (25) $$t=t_{1}+\Delta t$$

**Figure 5:**
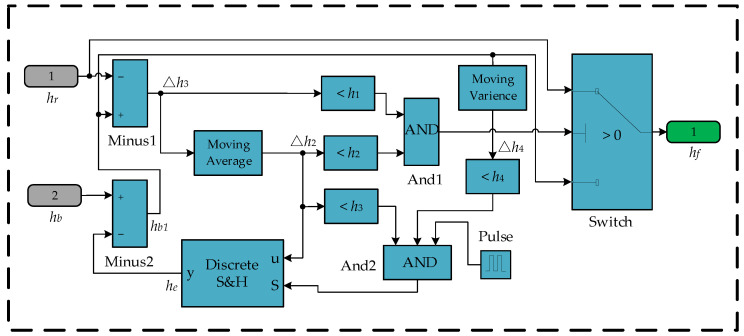
Altitude fusion method implementation in the Simulink environment.

The implementation of the altitude fusion method in Simulink is shown in Figure 5. The input data are *h_r_* and *h_b_*, while the output is the fused altitude *h_f_*. The Moving Average module is employed in the calculation of the average value of the altitude difference, the Moving Variance module is utilized in the computation of the variance of the flight altitude, the Discrete S&H module is responsible for storing the barometer measurement error, the Pulse module is used to generate pulses, and the Switch module is used for switching the output data.

The operational principle of altitude switching is as follows: The subtractor Minus2 subtracts the calibration value *h_e_* from *h_b_* to obtain the calibrated altitude *h_b_*_1_. The subtractor Minus1 then subtracts *h_r_* from *h_b_*_1_ to calculate the altitude difference Δ*h*_1_ between the laser rangefinder and the barometer. To improve the detection resolution for small objects, the Moving Average module smooths Δ*h*_1_, subsequently outputting the average value Δ*h*_2_. When Δ*h*_1_ < *h*_1_, it indicates that no large object is detected, satisfying switching condition (1). Similarly, when Δ*h*_2_ < *h*_2_, it indicates that no small object is detected, satisfying switching condition (2). When both conditions are met, the And1 module outputs 1, which controls the Switch module to output *h_r_*. Otherwise, the Switch module outputs *h_b_*_1_, thereby achieving the altitude-switching function.

The operational principle of altitude calibration is as follows: When Δ*h*_2_ is less than *h*_3_, it indicates very little difference between laser rangefinders and barometers in altitude measurements, satisfying the calibration condition (1). Similarly, when *D_h_* is less than *h*_4_, it indicates that the UAV altitude varies only slightly, satisfying calibration condition (2). Similarly, when the Pulse module outputs 1, it indicates that the calibration moment is reached, satisfying the calibration condition (3). When all three conditions are met, the And2 module outputs 1, which allows Δ*h*_2_ to be stored in the Discrete S&H module, updates *h_e_,* and implements the barometer calibration function.

### 3.2. Positioning-Filtering Algorithm Based on UKF

In the second section of this paper, the structure of the dual-anchor model is briefly introduced, and the calculation coordinate formula for the three directions (x, y, z) is derived. And the motion model of the dual-anchor UAV can be established. The mathematical model can be described as follows:

(26) $$\left\{\begin{array}{l} x_{t+1}=f(x_{t})+w_{t},w_{t}\sim N(0,Q_{t})\\ z_{t}=g(x_{t})+n_{t},n_{t}\sim N(0,R_{t}) \end{array}\right.$$

where f(·) and g(·) are the state transfer and observation functions, respectively. ***x****_t_* and ***z****_t_* are the system state and observation vectors, respectively. ***w****_t_* and ***n****_t_* are the system process noise and the measurement noise, respectively, which are two distinct, independent types of noise. Furthermore, they both follow a normal distribution with covariances **Q***_t_* and **R***_t_*, respectively.

The motion of a certain axis of the UAV is analyzed by selecting position *p*, velocity *v*, and acceleration *a* as the system states, and using the system function for description, as shown in the following equation:

(27) $$f:\left\{\begin{array}{l} p_{t+1}=p_{t}+\Delta tv_{t}+0.5\Delta t^{2}a_{t}+w_{t,1}\\ v_{t+1}=v_{t}+\Delta ta_{t}+w_{t,2}\\ a_{t+1}=a_{t}+w_{t,3} \end{array}\right.$$

Based on the above Equation (27), it is evident that system function f(·) is linear. By utilizing matrix representation, this system function can be expressed as follows:

(28) $$\left[\begin{array}{c} p_{t+1}\\ v_{t+1}\\ a_{t+1} \end{array}\right]=A\left[\begin{array}{c} p_{t}\\ v_{t}\\ a_{t} \end{array}\right]=\left[\begin{array}{ccc} 1 & \Delta t & 0.5\Delta t^{2}\\ 0 & 1 & \Delta t\\ 0 & 0 & 1 \end{array}\right]\left[\begin{array}{c} p_{t}\\ v_{t}\\ a_{t} \end{array}\right]+\left[\begin{array}{c} w_{t,1}\\ w_{t,2}\\ w_{t,3} \end{array}\right]$$

Extending the system to the *x-*, *y-*, and *z*-axes, the system vector is expanded as follows:

$$x={\left[\begin{array}{ccccccccc} p_{x} & p_{y} & p_{z} & v_{x} & v_{y} & v_{z} & a_{x} & a_{y} & a_{z} \end{array}\right]}^{T}$$

The extended system transfer matrix A is shown as follows:

$$A=\left[\begin{array}{ccc} I_{3\times 3} & \Delta t*I_{3\times 3} & 0.5\Delta t^{2}*I_{3\times 3}\\ 0_{3\times 3} & I_{3\times 3} & \Delta t*I_{3\times 3}\\ 0_{3\times 3} & 0_{3\times 3} & I_{3\times 3} \end{array}\right]$$

where **I** is the unit matrix and **0** is the zero matrix.

*L_a_*, *l_b_*, *h_f_*, *as_x_*, *as_y_,* and *as_z_* are selected to form observation vector ***z*** as shown in the following equation:

$$z={\left[\begin{array}{cccccc} l_{a} & l_{b} & h_{f} & as_{x} & as_{y} & as_{z} \end{array}\right]}^{T}$$

where *l_a_* and *l_b_* are the distances between the UAV and anchors A and B, respectively, and their relationship with the state vector is as follows:

(29) $$\left\{\begin{array}{l} l_{a}=\sqrt{p_{x}^{2}+p_{y}^{2}+p_{z}^{2}}\\ l_{b}=\sqrt{{\left(a-p_{x}\right)}^{2}+p_{y}^{2}+p_{z}^{2}} \end{array}\right.$$

where *h_f_* is the observed flight altitude of the UAV, which is derived using an altitude fusion method, and the observed accelerations of the UAV along the *x-*, *y-*, and *z*-axes are represented by *as_x_, as_y_*, and *as_z_*, respectively. They are obtained through measurements from the accelerometer. Therefore, observation function g(·) can be defined as follows:

(30) $$g:\left\{\begin{array}{l} l_{a}=\sqrt{p_{x}^{2}+p_{y}^{2}+p_{z}^{2}}+n_{1}\\ l_{b}=\sqrt{{\left(a-p_{x}\right)}^{2}+p_{y}^{2}+p_{z}^{2}}+n_{2}\\ h_{f}=p_{z}+n_{3}\\ as_{x}=a_{x}+n_{4}\\ as_{y}=a_{y}+n_{5}\\ as_{z}=a_{z}+n_{6} \end{array}\right.$$

The EKF is frequently utilized in nonlinear systems and linearized nonlinear models using partial derivatives and first-order Taylor series expansion, disregarding quadratic and higher-order terms [35]. This process engenders errors, particularly in the case of strong nonlinearities and high dimensions. The UKF is a discrete-time filtering algorithm that employs the untraced transform (UT) to address the propagation issue of nonlinear functions in the state estimation of nonlinear systems, and the filtering effect is better than the EKF in strongly nonlinear systems [36]. The observation vector of the proposed dual-anchor model is nonlinear. In addition, the UKF has been proved to have more advantages than the EKF when dealing with prominent noise [37], and the proposed highly fused switching altitude data can be regarded as encountering sudden noise changes, so using the UKF will obtain better filtering effect.

Assuming **x** has a mean $\bar{x}$ and covariance **P***_x_*, a set of 2*n* + 1 (*n*, the state dimension) sigma points can be chosen, which is shown in Equation (31):

(31) $$\left\{\begin{array}{c} \chi_{0}=\bar{x}\\ \chi_{i}=\bar{x}+{\left(\sqrt{(n+\lambda )P_{x}}\right)}_{i},i=1,2,\cdots ,n\\ \chi_{i}=\bar{x}-{\left(\sqrt{(n+\lambda )P_{x}}\right)}_{i},i=n+1,n+2,\cdots ,2n \end{array}\right.$$

where n = 9, and λ = 3–n. The mean weight and covariance weight of these points are denoted as W_i_^(*m*)^ and W_i_^(*c*)^, respectively, and the following formula determines their values:

(32) $$\left\{\begin{array}{c} W_{0}^{m}=\lambda /\left(n+\lambda \right)\\ W_{0}^{c}=\lambda /\left(n+\lambda \right)+1-\alpha^{2}+\beta \\ W_{i}^{m}=W_{i}^{c}=\lambda /\left[2\left(n+\lambda \right)\right],i=1,2,\cdots ,2n \end{array}\right.$$

where α = 0.001, and β = 2. The set of points can approximate the Gaussian distribution of the state vector **x**. All the points would be nonlinear transformed in this step according to the nonlinear function. The results can be expressed as follows:

$$Y_{i}=j(\chi_{i})$$

The distribution of **y** = j(**x**) can be approximately revealed by the set of sigma points {Y*_i_*}. After weighted calculation, the mean and covariance of **y** are obtained as follows:

(33) $$\left\{\begin{array}{c} \bar{y}=\sum_{i=0}^{2n}W_{i}^{m}Y_{i}\\ P_{y}=\sum_{i=0}^{2n}W_{i}^{c}(Y_{i}-\bar{y}){\left(Y_{i}-\bar{y}\right)}^{T} \end{array}\right.$$

In the standard UKF algorithm, the nonlinear transformation of system function f(·) and observation function g(·) is carried out, respectively. The above analysis indicates that f(·) is linear, and g(·) is nonlinear. So only a nonlinear transformation of g(·) is needed, thus reducing the amount of computation. The process of implementing a UKF in the dual-anchor positioning model can be summarized as follows:

**Step 1:** For initialization, in the vector $x_{0}$, the elements *p_x_*_0_, *p_y_*_0_, and *p_z_*_0_ are calculated using the measurements *l_a_*_0_, *l_b_*_0_, and *h_f_*_0_ and the Formula (6), while the values of the other elements are set to 0.

(34) $${\bar{x}}_{0}=E(x_{0}),P_{0}=E[(x_{0}-{\bar{x}}_{0}){\left(x_{0}-{\bar{x}}_{0}\right)}^{T}]$$

**Step 2:** At the time step t, the sigma point set is generated, and the mean and the covariance of the state are predicted using the transformation matrix **A** as follows:

(35) $$\left\{\begin{array}{c} x_{i,t+1|t}=A\chi_{i}\\ {\hat{x}}_{t+1|t}=\sum_{i=0}^{2n}W_{i}^{m}x_{i,t+1|t}\\ P_{t+1|t}=\sum_{i=0}^{2n}W_{i}^{c}\left(x_{i,t+1|t}-{\hat{x}}_{t+1|t}\right){\left(x_{i,t+1|t}-{\hat{x}}_{t+1|t}\right)}^{T}+Q_{t} \end{array}\right.$$

**Step 3:** A new set of sigma points $\chi_{i}^{*}$ is generated and the mean, variance, and covariance of the observations are predicted as follows:

(36) $$\left\{\begin{array}{c} z_{i,t+1|t}=g\left(\chi_{i}^{*}\right)\\ {\hat{z}}_{t+1|t}=\sum_{i=0}^{2n}W_{i}^{m}z_{i,t+1|t}\\ P_{zz,t+1|t}=\sum_{i=0}^{2n}W_{i}^{c}(z_{i,t+1|t}-{\hat{z}}_{t+1|t}){\left(z_{i,t+1|t}-{\hat{z}}_{t+1|t}\right)}^{T}+R_{t+1}\\ P_{xz,t+1|t}=\sum_{i=0}^{2n}W_{i}^{c}(x_{i,t+1|t}-{\hat{x}}_{t+1|t}){\left(z_{i,t+1|t}-{\hat{z}}_{t+1|t}\right)}^{T} \end{array}\right.$$

**Step 4:** The time step is updated and the Kalman gain calculated as follows:

(37) $$K=P_{xz,t+1|t}*P_{zz,t+1|t}^{-1}$$

**Step 5:** The system state and covariance matrix are updated as follows:

(38) $$\left\{\begin{array}{c} {\hat{x}}_{t+1}={\hat{x}}_{t+1|t}+K(z_{t+1}-{\hat{z}}_{t+1|t})\\ P_{t+1}=P_{t+1|t}-KP_{zz,t+1|t}K^{T} \end{array}\right.$$

To begin a new round of calculations, return to Step 2. The outputs of the algorithm, *p_x_*, *p_y_*, and *p_z_*, are selected. This is achieved by multiplying ${\hat{x}}_{t}$ and matrix **C**, and the following equation is used:

(39) $${\left[\begin{array}{ccc} p_{x} & p_{y} & p_{z} \end{array}\right]}^{T}=C{\hat{x}}_{t+1}=\left[\begin{array}{cc} I_{3\times 3} & 0_{6\times 3} \end{array}\right]{\hat{x}}_{t+1}$$

## 4. Methods

In order to evaluate the proposed dual-anchor model correctly, the positioning errors of the two models are analyzed first. The proposed altitude fusion algorithm and UKF algorithm are then tested in simulated environments, respectively. Finally, a physical test platform is built, and static, dynamic, and comprehensive tests are carried out in real environments, respectively. The experimental setup and evaluation method adopted are described in the following sections.

### 4.1. Simulations Methods 

#### 4.1.1. Simulation of Positioning Errors

To visually analyze the positioning error of the dual- and three-anchor models, the variations in positioning error along the *x-* and *y*-axes are presented in graphical form. To compare positioning errors within the simulation environment, the coordinates of anchors A and B were configured to be identical across both positioning models. The coordinates of anchors A and B were thus set to A(0, 0, 0) and B(0, 10, 0), respectively. Subsequently, the coordinate of anchor C for the three-anchor positioning model was set to C(0, 2, 8). The area designated for positioning was defined as a square. As discussed in Section 2.3, the positioning area should be situated at a specific distance from the coordinate axes to achieve enhanced positioning precision. Consequently, the location area was set to be 2 m away from both the *x-* and the *y*-axes, with its four vertices located at (2, 2), (2, 10), (10, 10), and (10, 2). The simulation set the ranging error of the UWB to 10 cm. Similarly, the TF-Mini laser rangefinder [38], a commonly utilized ground rangefinder produced by BeiWake, exhibits a maximum ranging error of 2 cm at short distances. Therefore, the altitude measurement error of the laser rangefinder was also set to 2 cm in the simulation. The UAV was set to fly in a plane 2 m from the ground. The simulation results are presented in Section 5.1.1.

#### 4.1.2. Simulation of the Altitude Fusion Method

The simulation of the altitude fusion method comprises two distinct aspects: first, ascertaining the correctness of the logic employed for altitude data switching and calibration; and second, evaluating the efficacy of altitude fusion. The methodology used for assessing the UAV comprises the following steps: First, the UAV was allowed to take off and flew for a designated period above an area devoid of obstacles. Subsequently, the UAV flew to an area containing impediments, such as boxes or slopes, where it remained for a designated period before departing the obstacle area. Finally, it flew out of the obstacle area and continued flying. The simulation results are presented in Section 5.1.2.

#### 4.1.3. Simulation of the Positioning-Filtering Algorithms

The function of the position-filtering algorithm is to reduce the positioning error by fusing the acceleration of the UAV. To evaluate the effectiveness of the positioning-filtering algorithm, the simulation was carried out as follows: The UAV is designated to take off, fly, and land along a predetermined route. The UAV’s state vector was calculated at each point along this route. The observation vector was then derived using Equation (30), incorporating the state vector with added observation noise. The original position coordinates were computed using Equation (6) and the observation vectors. These were further refined by applying the UKF algorithm filtered positioning coordinates. Finally, the course, original coordinates, and filtered coordinates were plotted and analyzed. The simulation results are presented in Section 5.1.3.

### 4.2. Experiments Methods

#### 4.2.1. UAV Experimental Platform

**Figure 6:**
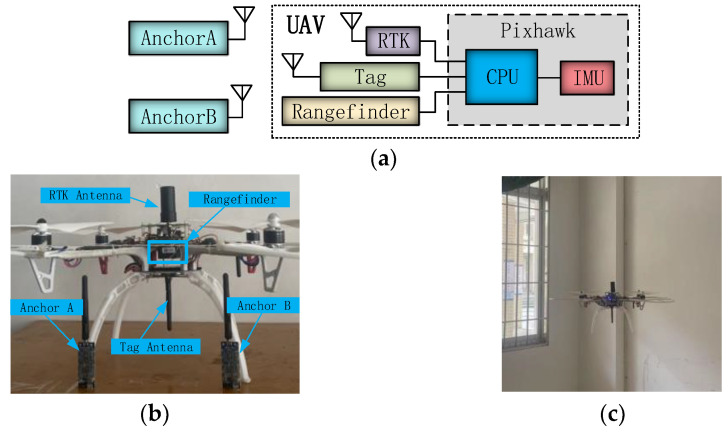
UAV experimental platform diagram: (**a**) UAV experimental platform structure diagram; (**b**) physical diagram of UAV experimental platform; and (**c**) UAV indoor flight.

The UAV experimental platform is shown in Figure 6. Figure 6a shows the structure diagram of the platform, including the UWB anchors and the UAV. The part unrelated to positioning is not drawn. In both anchors A and B, a DW1000 model measuring chip was employed, exhibiting a measurement error of less than 10 cm and an effective measuring distance of 100 m. The UAV was equipped with a UWB tag, a laser rangefinder, and a flight controller. The UWB tag also incorporated the DW1000 chip, ensuring performance parity with the UWB anchors. The laser rangefinder employed was a TF-Mini, which has a maximum measuring distance of 10 m. The model of the flight controller system was a Pixhawk, comprising a CPU and an IMU module. The IMU consists of an accelerometer, a barometer, and so on. The flight controller reads distance data between the UAV and anchors from the UWB tag; altitude information from the laser rangefinder; and acceleration, atmospheric pressure, and temperature data from the IMU module to compute the UAV position accurately. The RTK is used to measure the actual position of the UAV. Figure 6b shows the actual UAV testing platform, while Figure 6c depicts an indoor flight scenario for the UAV.

#### 4.2.2. Static Positioning Experiment and Analysis

To analyze the static positioning performance of the system, static positioning experiments were conducted in this study. The UAV was placed stationary in the positioning area, and the positioning data were recorded for a period of time. Anchors A and B were deployed at coordinates (0, 0, 0) and (4, 0, 0), respectively. The UAV was sequentially placed at the following coordinates: a(0.8, 0.8, 0), b(4, 0.8, 0.5), c(2.4, 2, 1.0), d(0.8, 4, 1.5), and e(4, 4, 2.0). The experiment results are presented in Section 5.2.1.

#### 4.2.3. Dynamic Positioning Experiment

To analyze the dynamic positioning performance of the system, dynamic positioning experiments were conducted in this study. This experiment involved recording positioning data while the UAV flew along a predefined route, which was used to analyze the errors in dynamic positioning. Unlike static positioning, the actual position of the UAV during flight is unknown. To measure the true position of the UAV, a high-precision positioning device, RTK, was equipped on the UAV. The horizontal positioning accuracy of RTK is approximately 2–3 cm, which is an order of magnitude higher than the UWB positioning accuracy, making it a suitable reference for the UAV’s actual horizontal position. However, the vertical positioning accuracy of RTK is about 5cm, which is worse than that of laser rangefinders. Therefore, the RTK altitude data were not used.

Due to RTK relying on GPS signals, testing must be conducted in an outdoor setting. During the experiment, the UAV was set to fly outdoors along a circular flight route with a center coordinate of (6, 6, 1) and a diameter of 2 m at a speed of 50 cm/s. The RTK positioning data, as well as the original and filtered positioning data, were recorded in real time during the flight. The experiment results are presented in Section 5.2.2.

#### 4.2.4. Comprehensive Experiment

**Figure 7:**
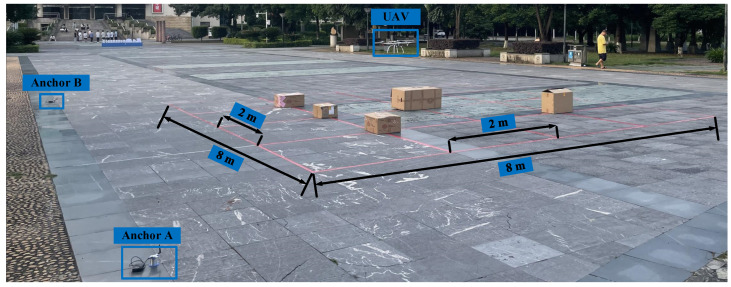
Comprehensive experimental site scene.

In the comprehensive experiment, the UAV flew along a complex route in a challenging environment to simulate indoor flight. Throughout the flight, both the positioning data and RTK data were recorded to plot the UAV’s trajectory and subsequently analyze and compare the positioning data. The comprehensive test site was located at the Guangxi University of Science and Technology square, with a horizontal flight area of 8 m × 8 m. Within this area, 10 straight flight routes were evenly distributed, forming 16 squares of 2 m × 2 m each. The flights were conducted at altitudes of 1 and 2 m, with a set flight speed of 50 cm/s. Figure 7 illustrates the test scenario. It depicts two anchors deployed on the ground, designated as A and B, with a distance of 12 m between them in a linear arrangement. The flight routes were marked with red tape, and to simulate an indoor flight environment, several boxes of varying heights were randomly placed on the ground. Since RTK positioning accuracy and UAV flight performance were strongly affected by weather conditions, testing was scheduled for sunny or breezy days. The experiment results are presented in Section 5.2.3.

## 5. Results and Discussion

The experimental results obtained are presented and discussed in this section, as outlined in the previous section. Two types of measurements were conducted: The first group was performed in a simulated environment to assess the feasibility of the proposed dual-anchor model. The second set of experiments was carried out in a real-world environment, where both static and dynamic along with comprehensive performances of the proposed dual-anchor model, were qualitatively assessed.

### 5.1. Simulation Results

#### 5.1.1. Results of Positioning Errors Simulation

**Figure 8:**
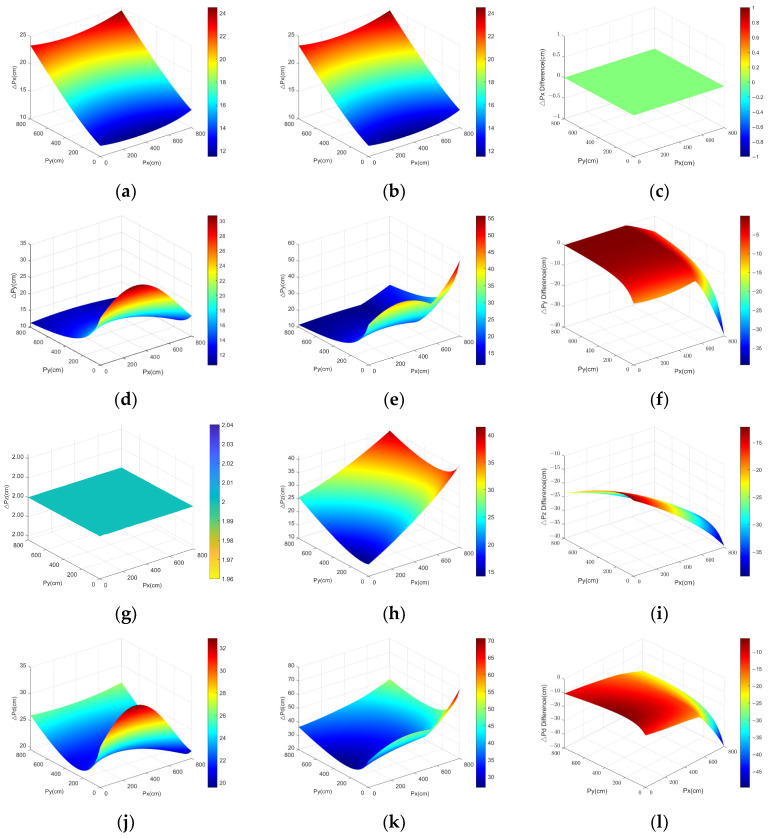
Positioning error surface plot for dual- and three-anchor models: Column 1 contains subgraphs (**a**,**d**,**g**,**j**) showing the positioning errors of the dual-anchor model. Column 2 contains subgraphs (**b**,**e**,**h**,**k**) showing the positioning errors of the three-anchor model. Column 3 contains subgraphs (**c**,**f**,**i**,**l**) showing the discrepancy between the dual- and three-anchor positioning errors. Line 1 contains subgraphs (**a**–**c**) showing the positioning errors of the *x*-axis △*p_x_*. Line 2 contains subgraphs (**d**–**f**) showing the positioning errors of the *y*-axis △*p_y_*. Line 3 contains subgraphs (**g**–**i**) showing the positioning errors of the *z*-axis △*p_z_*. Line 4 contains subgraphs (**j**–**l**) showing the positioning errors of the spatial distances △*p_d_*.

The parameters mentioned in Section 4.1.1 were then applied to Equations (10) and (12), respectively, and a simulation was performed to obtain the positioning error surface map, whose coordinates were translated 200 cm from the *x-* and *y*-axes, respectively, so that the location region starts at the origin, as illustrated in Figure 8.

Here, △*p_d_* was calculated as follows:

(40) $$\Delta p_{d}=\sqrt{\Delta p_{x}^{2}+\Delta p_{y}^{2}+\Delta p_{z}^{2}}$$

As shown in Figure 8, we separately describe the errors along the *x-*, *y-*, and *z*-axes and the distance errors to enable a more comprehensive analysis and comparison of the error distribution patterns of the two positioning models. For positioning error △*p_x_* along the *x*-axis, both models exhibit a similar pattern: △*p_x_* is influenced little by *p_x_* but has a stronger relationship with *p_y_*. As *p_y_* increases, △*p_x_* also increases, with a maximum value of 24.5 cm for both models.

For positioning error △*p_y_* along the *y*-axis, △*p_y_* is also influenced little by *p_x_* but is more closely related to *p_y_*. As *p_y_* decreases, △*p_y_* also increases, with the maximum △*p_y_* being 30.7 and 55.6 cm for the dual- and three-anchor positioning models, respectively.

For positioning error △*p_z_* along the *z*-axis, the △*p_z_* of the dual-anchor positioning model is not influenced by *p_x_* or *p_y_* and is simply the inherent error of the rangefinder, which is 2 cm. In contrast, the △*p_z_* of the three-anchor model is significantly influenced by *p_x_* and *p_y_*, with a maximum error reaching 41.5 cm.

Considering the positioning distance error △*p_d_*, the △*p_d_* of the dual-anchor positioning model is influenced little by *p_x_* but is more strongly affected by *p_y_*, with a maximum value of 32.6 cm. Compared with dual-anchor positioning, the △*p_d_* of the three-anchor model is more strongly influenced by both *p_x_* and *p_y_*, with a maximum value of 70.6 cm.

△*p_x_* shows the same pattern in the different surface plot. Moreover, the discrepancies in △*p_y_*, △*p_z_*, and △*p_d_* on the surface plot are nearly negative, suggesting that, in general, the positioning error of the dual-anchor model is consistently smaller than that of the three-anchor model.

Following the alteration in the UAV flight altitude to 1 m and 3 m, the simulation was executed, resulting in the acquisition of the maximum positioning errors at varying altitudes, as shown in Table 1. The data indicate that the error of dual-anchor positioning remains relatively constant with changes in flight altitude. By contrast, the positioning error of the three-anchor system increases with altitude, particularly with a notable change in Δ*p_y_*. Through analysis of the data from the figures and tables, it is clear that the positioning errors of the dual-anchor system are smaller than those of the three-anchor system, indicating that the dual-anchor system has higher positioning accuracy, particularly in the *z*-axis where the error is smaller. This is because the altitude data are obtained directly through measurement, as opposed to being calculated.

Remarks: It should be noted that the error simulation experiment in this section selects the maximum error of the laser rangefinder and UWB as the simulation input. It can thus be defined that the resulting error simulation is maximum error-bound. It should be noted, however, that the ranging error of the laser rangefinder and UWB typically exhibits fluctuations within the maximum error range and does not necessarily correspond to the maximum error value. Consequently, the error simulation results presented in this section are considerably larger than the subsequent physical measurement results.

**Figure 9:**
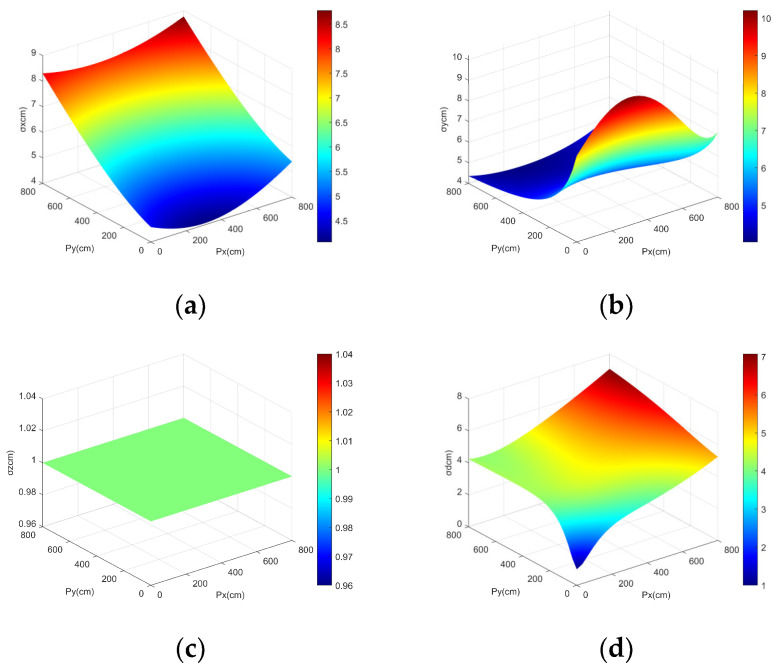
Standard deviation surface plot for dual-model system: (**a**) Standard deviation surface on the *x*-axis. (**b**) Standard deviation surface on the *y*-axis. (**c**) Standard deviation surface on the *z*-axis. (**d**) Standard deviation surface on the spatial distances.

Subsequently, the standard deviation of the dual-anchor positioning model is simulated. Assuming that the standard deviation of *l_a_* and *l_b_* is 5 cm, and the standard deviation of the *h* is 1 cm, the simulation results for the standard deviation, as calculated according to Equations (15) and (16), are as shown in Figure 9.

#### 5.1.2. Results of the Altitude Fusion Method Simulation

**Figure 10:**
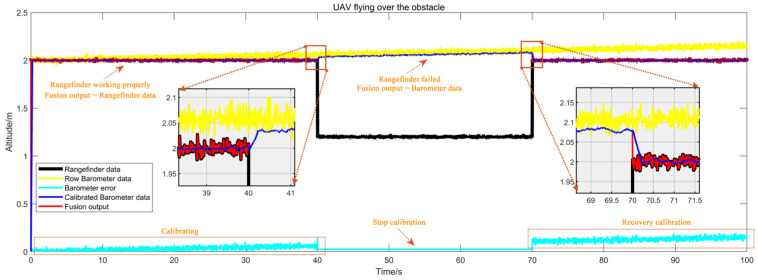
Simulation curves for obstacles as boxes.

The simulation method has been described in Section 4.1.2. Figure 10 illustrates the simulation of an encounter with a box during flight. In the initial 40 s following take-off, no obstacles were present below the UAV. During this period, the fusion method outputted data from the rangefinder while calibrating the barometer, thereby ensuring the continuous updating of the calibrated values of the barometric altimeter. As the duration approached 40 s, the error in the uncalibrated raw barometric data reached approximately 7 cm, while the discrepancy in the calibrated data was markedly reduced. At the 40-s mark, the UAV encountered a box below, resulting in a notable decrease in the rangefinder values and altitude change that exceeded the pre-established threshold. At this juncture, the barometer calibration value was temporarily halted, and the fusion method switched to output the barometer data. As the 70-s mark was approached, the halt of updates to the barometric altimeter error resulted in the error reaching 10 cm. At 70 s, upon the UAV’s departure from the obstacle area, the fusion method resumed outputting data from the rangefinder, and the barometer was calibrated once more. The discrepancy between the output values generated by the fusion method and the actual values remained below 10 cm throughout the flight, which is indicative of an effective fusion method.

**Figure 11:**
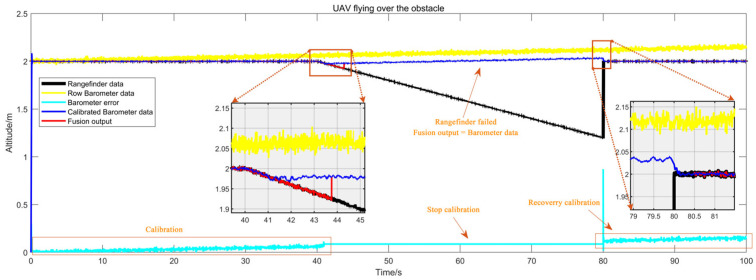
Simulation curves for obstacles as slopes.

In Figure 11, the obstacle was replaced by a slope while all other conditions remained identical to those in Figure 9. The sliding average filter smoothed the errors, thereby enhancing the resolution of the altitude detection. In the presence of an object with a slow altitude variation beneath the UAV, the altitude error would exceed the threshold after a certain period. At the 40 s mark, the UAV began to encounter the slope, resulting in a gradual decrease in the rangefinder measurement. The barometer calibration was temporarily halted at 41.5 s, after which the system was transitioned to barometer output at 43.8 s. At 80 s, the obstacle disappeared, allowing for the resumption of barometer calibration.

#### 5.1.3. Results of the Positioning-Filtering Algorithm Simulation

**Figure 12:**
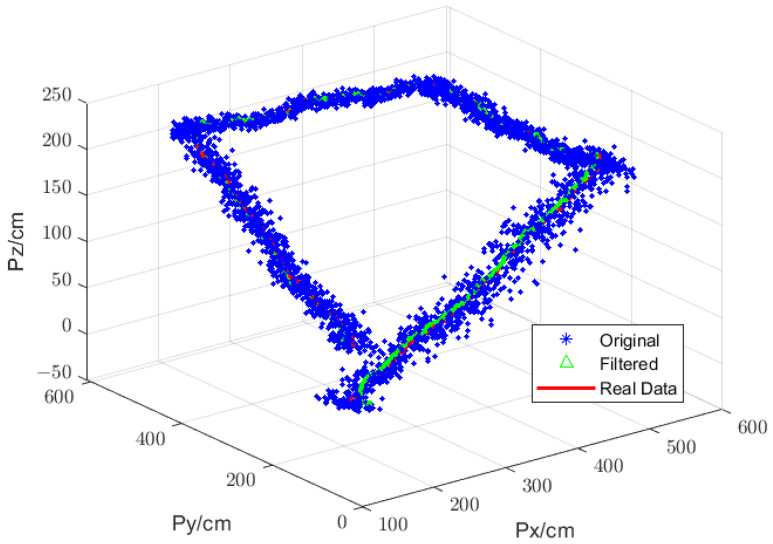
Simulation of the positioning-filtering algorithm.

The simulation method has been described in Section 4.1.3. The simulation results are shown in Figure 12, where it can be seen that both the original and filtered positioning coordinates are distributed on either side of the flight route, with the original positioning coordinates being further away from the flight route. In order to evaluate the performance of positioning, the root-mean-square error (RMSE) of the positioning of the 3D plane is calculated. The following formula is utilized to facilitate this calculation:

(41) $$RMSE=\sqrt{\frac{1}{n}\sum_{i=1}^{n}{\left(p_{d,i}-{\hat{p}}_{d,i}\right)}^{2}}$$

The distance error *Δpd* was calculated using Equation (41). After the calculations, the RMSE of the original coordinates was 17.6 cm, while the RMSE of the filtered coordinates was 11.2 cm. This indicates that the positioning-filtering algorithm effectively reduced the positioning errors.

### 5.2. Experiments Results

#### 5.2.1. Results of Static Positioning Experiment

**Figure 13:**
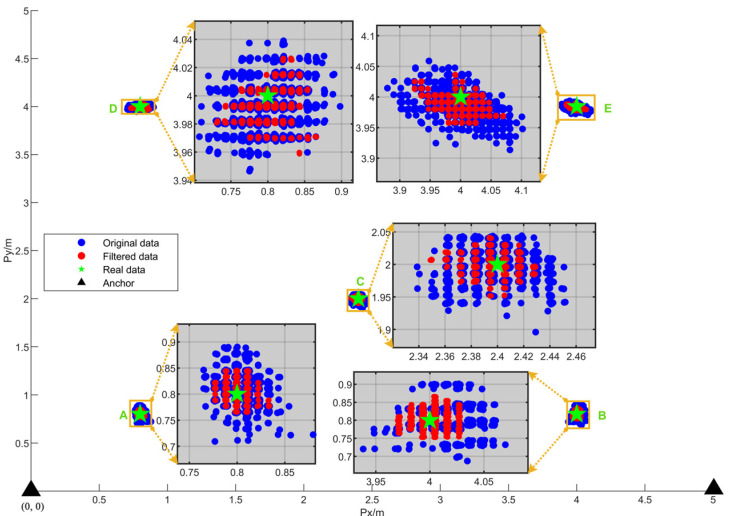
Static positioning experiment.

The experiment method has been described in Section 4.2.2. At each coordinate point, approximately 100 original and filtered data points were recorded as Figure 13. By zooming into the area where the UAV was located, the distribution of positioning data can be observed more clearly. The filtered data more closely approached the actual UAV coordinates than the original data, indicating that the positioning filter algorithm reduced measurement error.

**Figure 14:**
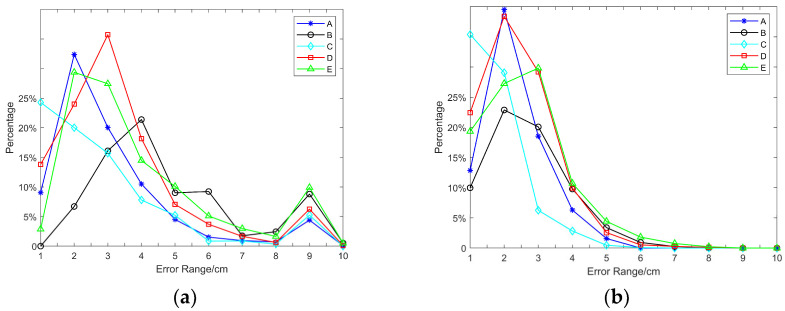
Positioning error comparison diagram: (**a**) The distribution curve of the original positioning error. (**b**) The distribution curve of the positioning error after filtering.

The positioning distance error, Δ*p_d_*, was divided into 10 intervals, (0, 1), (1, 2), …, (9, 10), which were labeled as intervals 1, 2, …, 10. The percentage of Δ*p_d_* within each interval was calculated and is presented as a line chart in Figure 14.

Figure 14a shows the distribution curve of the original positioning error, with the maximum error at each point being less than 10 cm and most errors being below 7 cm. Figure 14b displays the error curve after filtering, with the maximum error at each point being less than 8 cm and most errors falling below 5 cm, indicating that the positioning accuracy improved after filtering. Table 2 shows the RMSE for each point of original positioning and filtered positioning. After filtering, the positioning data were reduced by 1.5–3 cm, demonstrating the effectiveness of the positioning-filtering algorithm.

#### 5.2.2. Results of Dynamic Positioning Experiment

**Figure 15:**
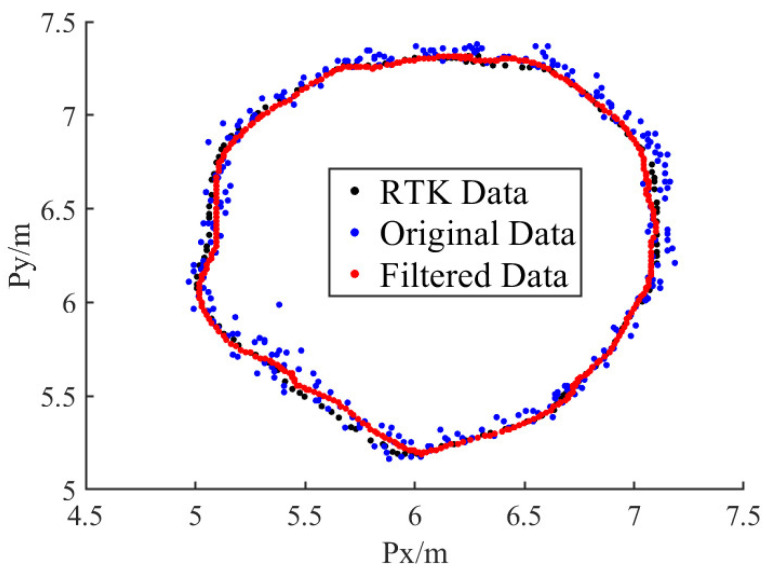
Dynamic positioning experiment.

The experiment method has been described in Section 4.2.3. The flight data are shown in Figure 15. Although the flight route was circular, the trajectory was not a standard circle, due to the influence of wind, which did not affect the analysis of the positioning errors, as the analysis referenced the RTK data rather than the predefined flight route. Figure 15 shows that both the original and the filtered data were distributed near the RTK data, with the original data being farther from the RTK data and exhibiting a random distribution pattern, while the filtered data were closer to the RTK data.

**Figure 16:**
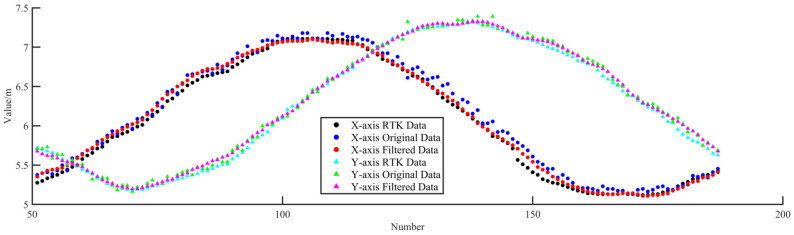
Data distribution on *x*- and *y*-axes.

The distribution of the data on the *x-* and *y*-axes is shown in Figure 16 and is similar to that of a sinusoidal wave, with the original and filtered data distributed near the RTK data and the filtered data closer to the RTK data.

The horizontal distance error was calculated using the following formula:

(42) $$\Delta p_{d}=\sqrt{\Delta p_{x}^{2}+\Delta p_{y}^{2}}$$

The RMSE of the *x*- and *y*-axes and that of △*p_d_* during dynamic positioning are shown in Table 3. The data show that the RMSE of △*p_d_* is 8.14 cm, indicating high positioning accuracy. After filtering the data, the RMSE of △*p_d_* is 4.93 cm, indicating that the accuracy is effectively improved by the UKF algorithm.

#### 5.2.3. Result of Comprehensive Experiment

**Figure 17:**
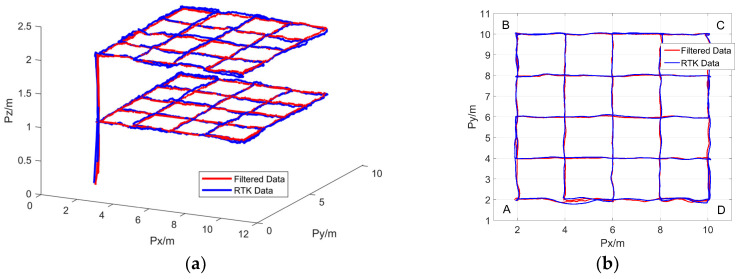
UAV flight trajectory: (**a**) 3D flight trajectory; (**b**) Horizontal flight trajectory: The A, B, C, and D are the four vertices of the outermost quadrilateral trajectory.

The experiment method has been described in Section 4.2.4. The 3D flight trajectory of the UAV is shown in Figure 17a, forming two layers of flight paths at altitudes of 1 and 2 m. The horizontal flight trajectory at an altitude of 1 m is depicted in Figure 17b. Throughout the flight, the UWB positioning data and RTK data nearly overlapped, indicating that the UWB positioning accuracy was very close to that of the RTK. To analyze the magnitude of positioning errors on different flight paths, four routes, AB, BC, CD, and DA, were selected for analysis, with the corresponding RMSE values presented in Table 4. As evidenced in Table 4, the lowest RMSE value was observed on path CD, with a recorded value of 8.1 cm, while the highest RMSE value was observed on path DA, with a recorded value of 14.2 cm. These test data indicate that the dual-anchor positioning model has decimeter-level positioning accuracy.

### 5.3. Comparison of Positioning Performance

The positioning performance of different types of positioning systems is compared in this section, and the single-anchor positioning model introduced in reference [30], the four-anchor positioning model introduced in reference [26], and the ten-anchor positioning system introduced in reference [18] are selected as the objects of comparison with the dual-anchor model. Despite the fact that the test data of each positioning system are obtained under independent test conditions, they are still comparable, and the positioning accuracy is shown in Table 5.

As demonstrated by the data, the dual-anchor and ten-anchor positioning models achieve 3D positioning, while the single-anchor and the four-anchor positioning systems attain 2D positioning. In terms of positioning accuracy, the accuracy of the dual-anchor model is better than the other three systems. A cost analysis, considering the number of UWB anchors utilized, reveals that the single-anchor system is the most economical, while the ten-anchor system is the most expensive.

## 6. Conclusions

This paper proposes an improved dual-anchor positioning model, which is based on the conventional UWB positioning principle, aiming to achieve decimeter-level 3D positioning with a reduced number of anchors. The utilization of the UAV sensor ensures the realization of 3D positioning with a mere two anchors. The adaptability of the model to operational environments is enhanced by addressing the issue of altitude measurement susceptibility to obstruction, thereby ensuring the reliability of the positioning system. The conclusions that can be drawn from this analysis are summarized as follows:

1. In the comprehensive experiment, the UAV successfully completed the flight of the complex route in the simulated indoor environment, thereby indicating the feasibility of the dual-anchor model in principle. The UAV demonstrated the capacity to accurately measure flight altitude when flying over the designated box, thereby validating the efficacy of the employed altitude fusion algorithm.
2. In the comparison of positioning accuracy, the maximum positioning RMSE of the dual-anchor model is 14.2 cm, which is at the same level as that of other multi-anchor models, indicating that it has decimeter-level positioning accuracy. In the dynamic experiment, the positioning RMSE decreased from 8.14 cm to 4.93 cm after applying the positioning-filtering algorithm, indicating that the UKF achieved a good filtering effect on the dual-anchor model.
3. Although the dual-anchor model is only a basic positioning unit, it can also be applied in multi-area positioning. In comparison with the multi-anchor model, the number of UWB anchors of the dual-anchor model can be reduced by approximately 33%. This reduction in anchors has two main benefits. Firstly, it results in a reduction in equipment cost. Secondly, it results in a reduction in deployment workload. The dual anchor model has a favorable cost-to-performance ratio, which is advantageous in practical applications.

The dual-anchor model is contingent upon precise altitude measurement and is well suited for utilization in indoor environments characterized by flat terrain, a limitation that is evident in other GNSS-denied environments, such as subterranean coal mines, where ensuring ground smoothness poses a significant challenge. In such contexts, it is imperative to devise alternative altitude measurement methods that can supplant the laser rangefinder, thereby enabling the deployment of the dual-anchor model.

## Figures and Tables

**Table 1:** Maximum positioning error of models.

Altitude	(Dual Anchor/Three Anchor)			
	*Δp_x_* (cm)	*Δp_y_* (cm)	*Δp_z_* (cm)	*Δp_d_* (cm)
1 m	24.2/24.2	28.4/33.5	2.0/39.5	30.5/48.4
2 m	24.5/24.5	30.7/55.6	2.0/41.5	32.6/70.6
3 m	24.9/24.9	33.8/94.3	2.0/50.1	36.0/108.0

**Table 2:** RMSE for 2D localization at each point (cm).

Position	A	B	C	D	E
Original	6.01	7.48	5.81	6.21	6.95
Filtered	3.96	4.46	3.77	3.96	5.48

**Table 3:** RMSE for localization (cm).

	*Δp_x_*	*Δp_y_*	*Δp_d_*
Original	8.14	4.93	8.14
Filtered	4.85	3.60	4.93

**Table 4:** RMSE of positioning error on different airlines (cm).

	*Δp_x_*	*Δp_y_*	*Δp_d_*
AB	3.9	7.8	8.6
BC	5.2	6.5	7.3
CD	7.8	4.0	8.1
DA	12.8	7.5	14.2

**Table 5:** Positioning accuracy comparison.

Number of Anchors	Additional Sensors	Fusion Algorithm	Dimensions	Accuracy (cm)
1	Odometry	KF	2D	21
2	Rangefinder, Barometer, Accelerometer	UKF	3D	14.2 (RMSE)
4	Camera	PF	2D	24 in 80%
10	/	/	3D	20 in 95%

## Data Availability

The original contributions presented in this study are included in the article material, and any further inquiries can be directed to the corresponding authors.
